# Understory growth of *Paris polyphylla* accumulates a reservoir of secondary metabolites of plants

**DOI:** 10.3389/fmicb.2024.1400616

**Published:** 2024-10-15

**Authors:** Xinru Yan, Dong Wang, Ao Zhang, Jing Xia, Jinlong Jiao, Murad Ghanim, Ou Xiaokun, Xiahong He, Rui Shi

**Affiliations:** ^1^Key Laboratory for Forest Resources Conservation and Utilization in the Southwest Mountains of China, Ministry of Education, International Ecological Forestry Research Center of Kunming, Southwest Forestry University, Yunnan, Kunming, China; ^2^Ministry of Education Key Laboratory for Microbial Resources, Yunnan University, Kunming, Yunnan, China; ^3^Department of Entomology, Institute of Plant Protection, Rishon LeZion, Israel

**Keywords:** *Paris polyphylla*, rhizome, secondary metabolites, steroidal saponin, plant flavonoids non-gras

## Abstract

*Paris polyphylla* is an important traditional medicinal plant of the Himalayan region. It is extensively used for the production of natural steroidal saponins and flavonoids. Although seed dormancy of wild plants can be broken to be artificially maintained and regenerated through micropropagation in the laboratory, the success of secondary metabolite production in higher quantities and the synthesis of superior plant metabolites have been very limited. In this study, we present differential metabolic profiling of *P. polyphylla* plants maintained for 8 years in natural and greenhouse conditions. Untargeted profiling of the metabolites through ultra-performance liquid chromatography-mass spectrometry/mass spectrometry (UPLC-MS/MS), followed by statistical analysis, identified secondary metabolites that were enriched in the naturally occurring plant roots compared with the greenhouse plant roots. A multivariate statistical analysis revealed the differential distribution of the compounds between the two groups. Overall, we identified 1,182 secondary metabolites, with 116 metabolites being differentially upregulated and 256 metabolites being downregulated. Moreover, 810 metabolites showed no significant variation under both growing conditions. The Kyoto Encyclopedia of Genes and Genomes (KEGG) analysis revealed that the naturally forest-grown *P. polyphylla* plants were significantly enriched in steroidal saponins, lipids, vitamins, flavonoids, and flavonols. An analysis of the top 10 differentially upregulated secondary metabolites indicated a significantly enriched quantity of spirost-5-en-3,12-diol and kaempferol synthesis pathways, which are known to reduce the effect of free radicals scavengers inside the cell. In addition, veratramine alkaloids were also enriched under natural conditions. Our findings indicated that naturally maintained *P. polyphylla* plants are suitable for the extraction of medicinally important compounds. Our study established a causal relationship between the metabolic composition of the roots and their natural growth condition. This study highlighted the importance of environmental conditions in the biosynthesis of secondary metabolites of plants.

## 1 Introduction

Any vegetation growing under a forest canopy is an integral component of forests (Smith et al., 2005; Helms, 1998). Understory vegetation plays a significant role in the maintenance of the ecosystem and biodiversity (Thomas et al., 1999). Furthermore, it interacts with several animal and insect species by acting as a great food and shelter source. In the majority of forests, only a small fraction of sunlight can cross the canopy to reach the understory. Understory plants of temperate forests experience unpredictable periods of solar radiation; therefore, they harbor a unique and diverse set of plants (Chazdon and Pearcy, 1991). Understory or ground vegetation is comprised of diverse species of plants and contributes to nutrient cycling and energy flows in forest plantations (Gilliam, 2007). Intense land utilization and conversion of natural forests into plantations are known to impact ground vegetation. During plantations, understory shrubs are generally removed, which can substantially damage the biodiversity, ultimately leading to species loss (Santana et al., 2011).

Understanding the dynamics of understory vegetation is important for sustainability and conservation of biodiversity. Understory vegetation in natural forests is characterized by unique layers of nutrient contents depending upon the type of plantation and environment. For example, decomposition of broad leaves can lead to higher nutrient accumulation, which can support vascular vegetation (Kumar et al., 2017). Coniferous plant litter produces an acidic environment, which supports the growth of small plants such as mosses and liverworts (Hart and Chen, 2008). However, due to the effects of shading, natural secondary forests have a very low level of disturbance in the shrub layers (Kumar et al., 2018; Sabatini et al., 2014). Previous studies have shown that light intensities and ratios can significantly change the composition of chemicals in certain understory plants (Wei et al., 2019). Controlled experiments conducted on understory plants showed that light spectrum is the key to photosynthesis and is directly responsible for the production and accumulation of carbohydrates (Li et al., 2018). Moreover, nutrient uptake and the metabolism of starch and soluble sugars (Zhao et al., 2019) are strengthened by adjustments to the lighting spectrum.

Flavonoids are natural compounds that are present in almost all plants. They are polyphenolic in nature and are considered low molecular weight secretary phytochemical secondary metabolites (Donadio et al., 2021). As the name indicates, secondary metabolites are synthesized in secondary pathways. They are needed in trace quantities (Roy et al., 2022). In addition to their role in plant development, secondary metabolites are an important part of our daily life. The secondary metabolites are synthesized by phenylpropanoids (Tajammal et al., 2022).

*Paris polyphylla* is a traditional medicinal plant naturally grown in China and the Indian subcontinent. Due to its century-old reputation as a traditional source of antibacterial, antifungal, antiparasitic, and anti-inflammatory medicine, different species in this group have been well-studied for their role as a medicine (Liu et al., 2012; Yin et al., 2018). Most of the important pharmacological components of traditional Chinese medicines (TCMs) have been isolated from their rhizome (Shi et al., 2004). Approximately 80% of the total number of active compounds are steroidal saponins [also called polyphyllins (PPs)] (Qin et al., 2012). There are several well-characterized polyphyllin (PP) compounds, namely, PP-I, PP-II, PP-VII, PP-H, and PP-D. These compounds are known to have anti-inflammatory, antitumor, anesthetic, antiseptic, and therapeutic properties (Chen et al., 2019; Lin et al., 2021). Important medically active compounds also include diosgenin and pennogenin (Wang et al., 2015). Currently, wild plants are the only source of rhizomes; however, efforts have been made to break the physiological dormancy for their growth in greenhouse conditions (Puwein and Thomas, 2022). Despite large-scale greenhouse cultivation, the quantity of active ingredients purified from *P. polyphylla* cannot satisfy the demands of the market. Due to the low reproductive rate and slow growth, wild plants cannot meet the demands of large pharmaceutical markets (Chen et al., 2007). In addition, the growth of rhizomes usually takes more than 7 years; therefore, more resources are needed to address the growing market demands.

With the help of omics strategies, several medicinal plants have been characterized at the genomic and protein levels (Christ et al., 2019; Morozova et al., 2009; Liu et al., 2016). However, the analysis of the metabolites of such plants has been very limited. It is well established that the older roots of *P. polyphylla* are more commercially valuable compared to the younger roots due to high saponin contents. In this study, we characterized the metabolome of *P. polyphylla* growing in both greenhouse and natural conditions. Using metabolic profiling, we found that the accumulation of secondary metabolites in understory covering is better than those in greenhouse planting. The present study aimed to evaluate the differential metabolites accumulated in *P. polyphylla* grown understory in a natural forest and in a greenhouse.

## 2 Materials and methods

### 2.1 Plant material

**Figure 1:**
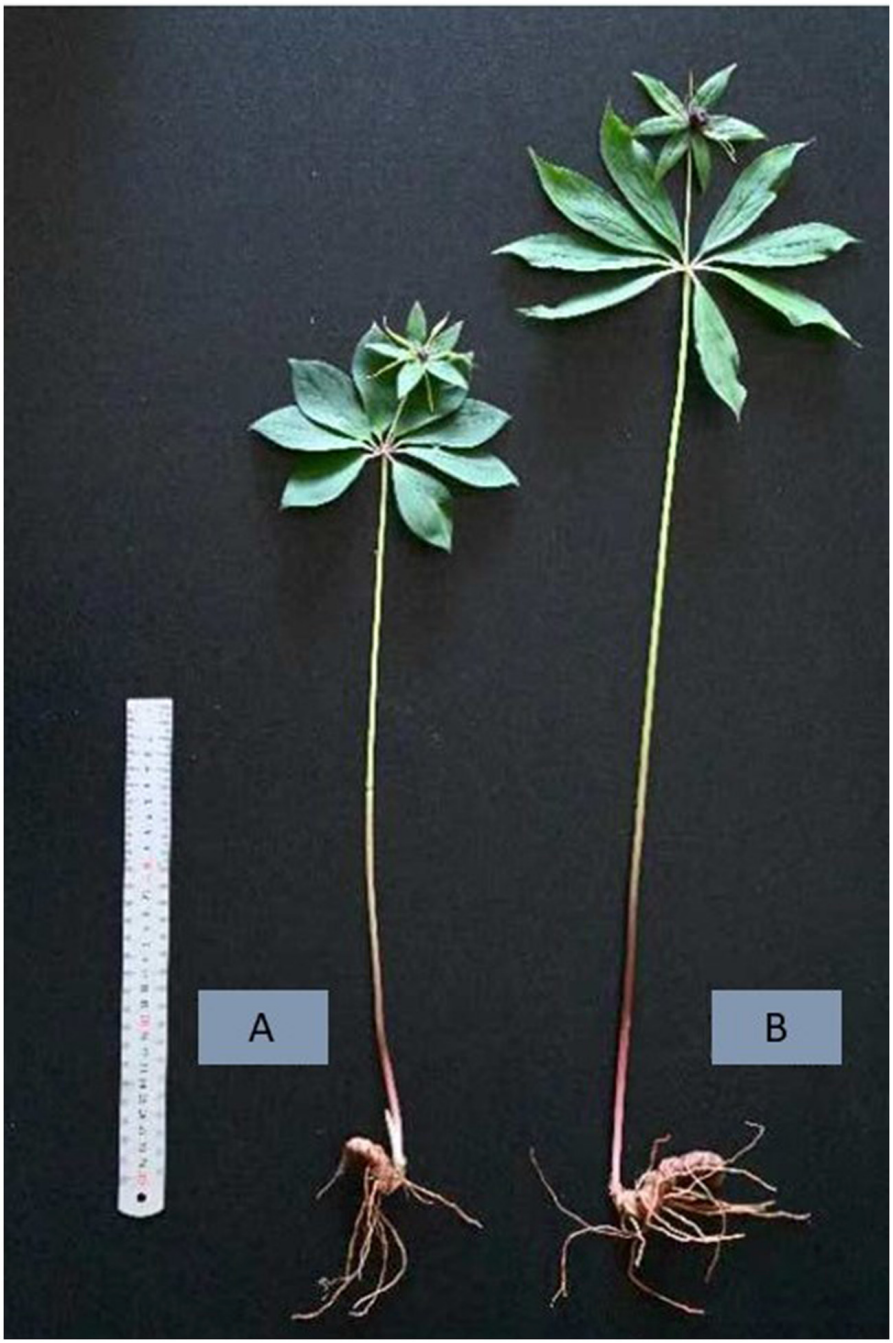
Physical appearance of *P. polyphylla* plants grown in a greenhouse **(A)** and the understory of the naturally occurring temperate forest **(B)**.

Three different samples (referred to as LPPR-1, LPPR-2, and LPPR-3) were collected from 8-year-old roots of *P. polyphylla* var. *yunnanensis* plants growing in the natural forests of Touzi Village, Zhehai Town, Huize county at 103.519° E longitude and 24.651° N latitude. In addition, three samples (referred to as PPR-1, PPR-2, and PPR-3) of 8-year-old *P. polyphylla* var. *yunnanensis* plants growing in the greenhouse of Southwest Forestry University China were collected for comparing the accumulation of secondary metabolites with the LPPR samples (Figure 1). The samples were cleaned and freeze-dried in a freeze-drying machine (Scientx-100F).

### 2.2 Sample extraction and UPLC-MS/MS analysis

The freeze-dried samples were converted into a fine powder using a grinder (MM 400, Retsch Gmbh, Germany). Approximately 50 mg of the powder from each sample was dissolved in 1.2 ml of 75% methanol (Merck). The metabolites in the samples of rhizomes were extracted with chromatographically pure methanol and 1% formic acid, as described in a previous study (De Vos et al., 2007). The samples were vortexed six times, each time for 30 s. The samples were centrifuged at 12,000 rpm for 3 min, and the supernatant was removed. The samples were filtered with a microporous membrane (a 0.22-μm pore size) and stored in a liquid phase for ultra-performance liquid chromatography-mass spectrometry/mass spectrometry (UPLC-MS/MS) analysis using UPLC (ExionLC™ AD, https://sciex.com.cn/), as described in a previous study (Chen et al., 2019). The chromatographic column (Agilent SB-C18 1.8 μm, 2.1 mm ^*^ 100 mm) was used for liquid phase extraction. The mass spectrometry conditions were adjusted as recommended by the manufacturer (ExionLC™ AD, https://sciex.com.cn/). Briefly, the mobile phase included phase A and phase B, where phase A was ultrapure water (0.1% formic acid) and phase B was acetonitrile (0.1% formic acid). In the beginning, the proportion of phase B was 5%, but later on, it linearly increased to 95%. The quantification of the metabolites was completed using multiple reaction monitoring (MRM). In the first step, the quadrupole first screened the precursor ions of the target substance and excluded ions corresponding to other molecular weight substances to eliminate the possibility of interference. The ion fragments were then selected through triple quadrupole filtration to eliminate interference from the non-target ions. The chromatographic peaks were integrated, and the mass spectrum peaks of similar metabolites were integrated and corrected (Fraga et al., 2010). Metabolite quantification was performed using the multiple reaction monitor (MRM) mode of the triple quadrupole mass spectrometry (MS). The MS data of different samples were integrated and corrected, as described previously (Fraga et al., 2010). The MS data were then processed using the software Analyst (Version, 1.6.3). The chromatogram of the total ion current (TIC) for the mixed quality control (QC) samples and MRM metabolites detected multiple peaks, which were used as a data assessment tool. Z-score (scaling for unit variance) was used to standardize the differential metabolite distribution.

### 2.3 Identification of differential metabolites

The detected metabolites (*n* = 1,182) were normalized for statistical analysis (Chen et al., 2013). Principal component analysis (PCA) was performed to depict the trend of the metabolome in individual samples, as described earlier (Chen et al., 2013). The R software package (www.r-project.org accessed on July 2023) was used as a statistical platform for the PCA. To characterize the metabolomic data, a multivariate statistical tool, orthogonal partial least squares discriminant analysis (OPLS-DA), combined with orthogonal signal correction (OSC), was used (Thévenot et al., 2015). A volcano plot was used to display the relative content difference of the metabolites in both samples. Briefly, the logarithmic value [fold change (FC) = Log^2^] and variable importance in projection (VIP) for the relative difference in the metabolites were used to draw the volcano plot. The significant level of difference (-log10 *p*-value) was calculated using the ordinate values in each case. Indeed, the larger coordinate values represented the larger significant differences.

The data used for the PCA analysis were also used for hierarchical cluster analysis (HCA) and Pearson correlation coefficient (PCC) analysis. The PCA and HCA analyses were performed using the R package integrated with ComplexHeatmap and presented as a heat map.

### 2.4 KEGG annotation and enrichment analysis

The differentially identified metabolites (from both the greenhouse and forest plants) were annotated using the Kyoto Encyclopedia of Genes and Genomes (KEGG) compound database (https://www.kegg.jp/kegg/compound/) and mapped to the KEGG pathway (https://www.kegg.jp/) (Kanehisa, 2000).

## 3 Results

### 3.1 Putative metabolite identification based on UPLC-MS/MS

**Figure 2:**
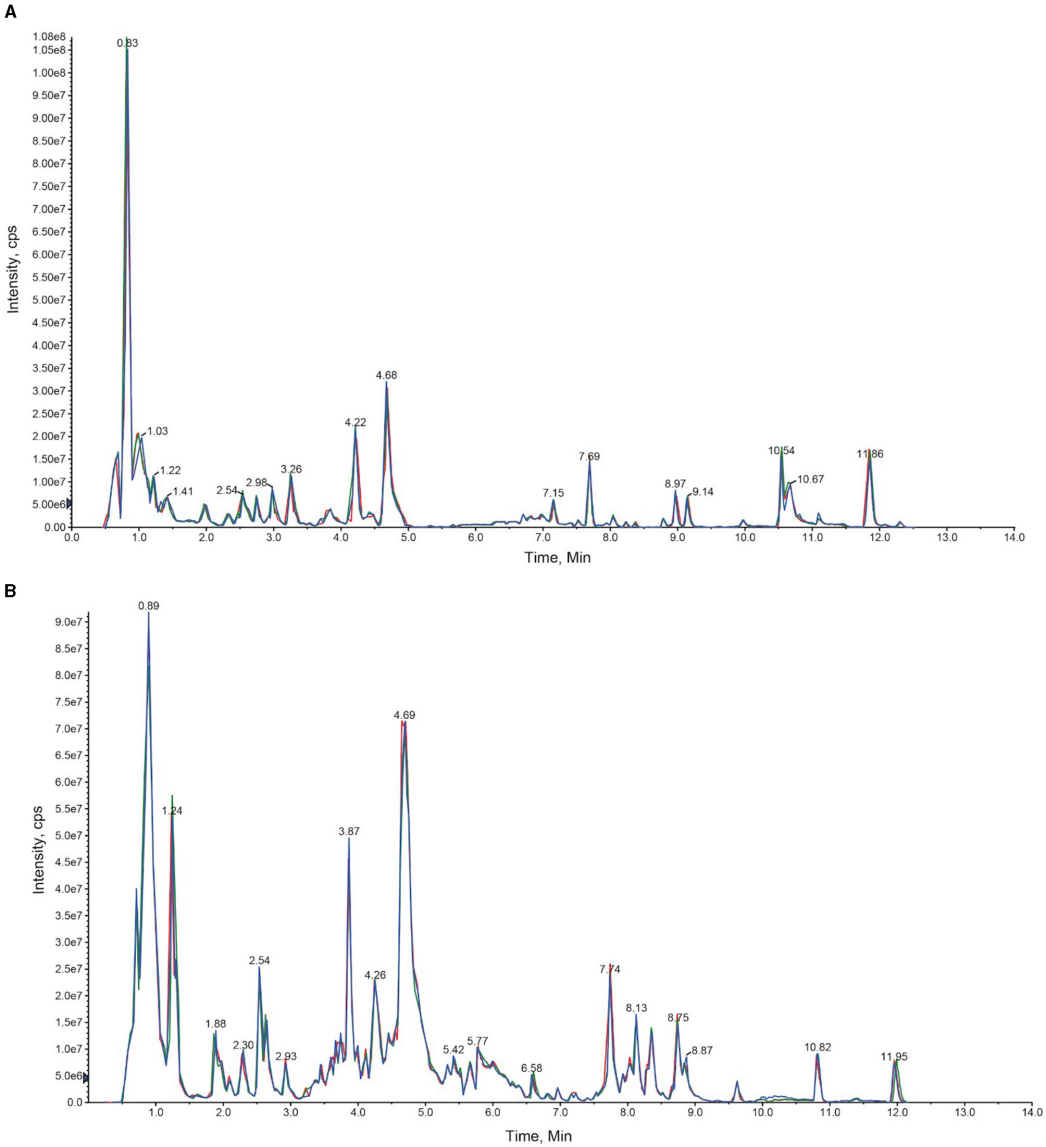
An overlay diagram of the total ion chromatogram (TIC diagram) detected by the mass spectrum of the QC samples. The retention time and consistency of peak intensities indicate the signal stability. **(A)** Represents the negative ion mode, and **(B)** represents the positive ion mode.

*Paris polyphylla* plants were divided into two broad categories, which included greenhouse plants and naturally occurring temperate forest plants in the Yunnan province of China. The extracted metabolites were first analyzed using the UPLC-MS/MS strategy. Through this analysis, total ion current (TIC) diagrams were obtained (Figure 2). The overlapping display of the TIC charts of the mass spectrometry detection and the analysis of the QC samples indicated a high level of signal stability and thus revealed the reliability of the data. The coefficient of variation (CV) value also revealed the stability and reliability of the data (Supplementary Figure S1).

**Figure 3:**
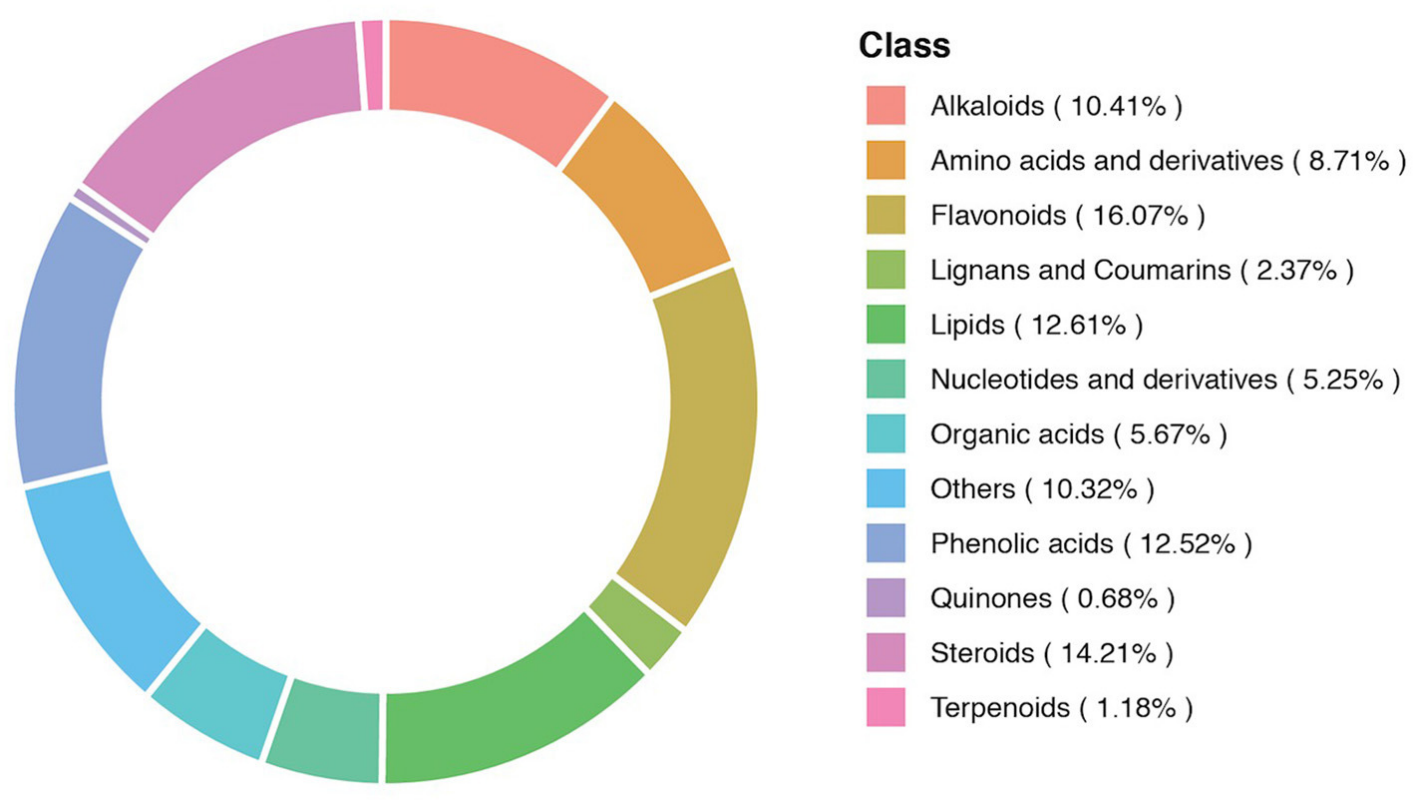
A donut diagram of the metabolite category composition. Each color represents a metabolite category, and the area of color indicates the proportion of that category.

The composition of the metabolites in different samples contained different categories and proportions of the metabolites. The composition analysis detected 12 different classes of the metabolites. These metabolites included alkaloids (10.41%), amino acids and derivatives (8.71%), and flavonoids (16.07%). Lignans and coumarins (2.37%), lipids (12.61%), nucleotides and derivatives (5.25%), organic acids (5.67%), phenolic acids (12.52%), quinones (0.68%), steroids (14.21%), terpenoids (1.18%), and others (10.32%). The metabolic composition ratio and distribution of major metabolites are presented in Figure 3.

### 3.2 Principal component analysis revealed overall variation among the samples

**Figure 4:**
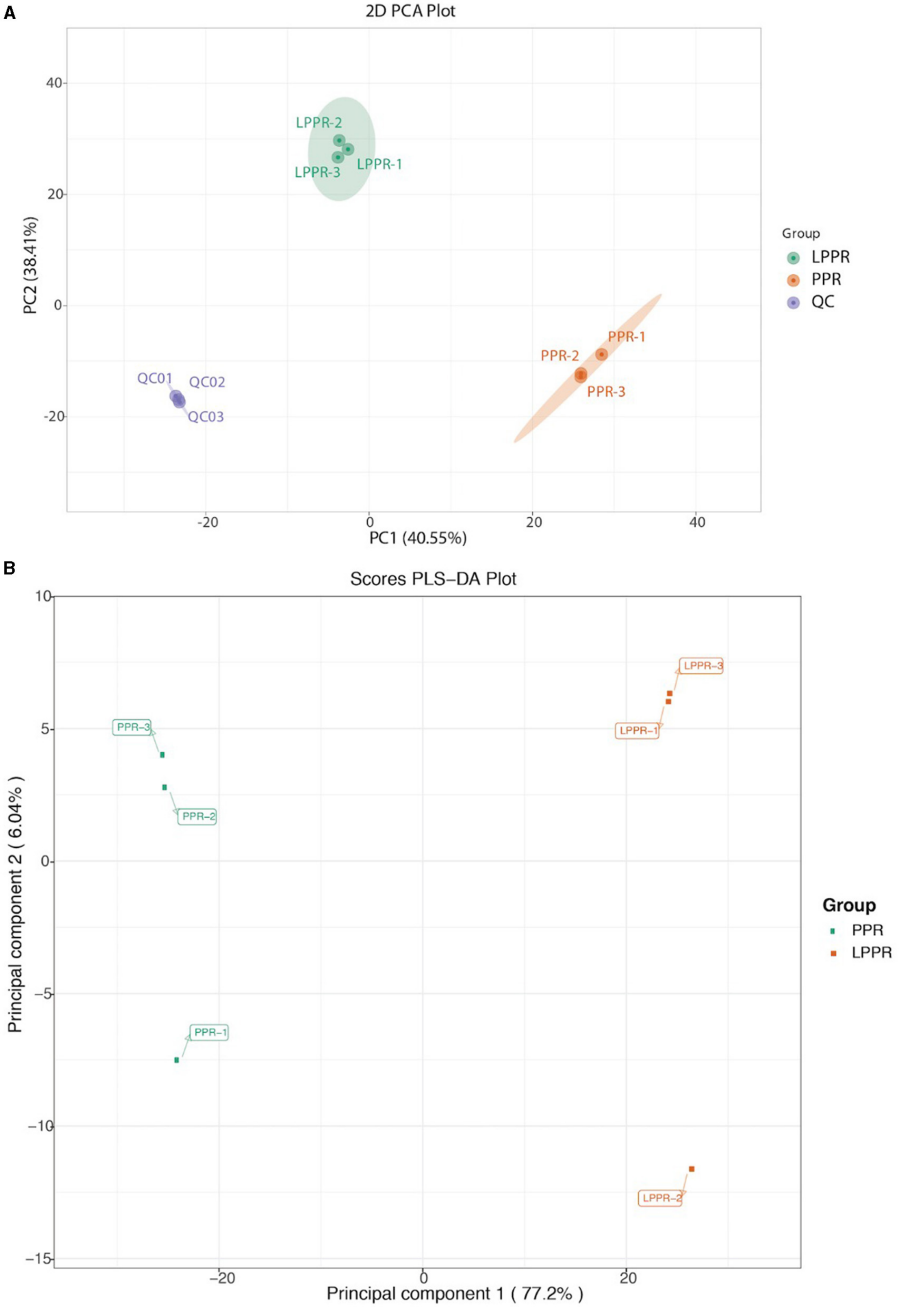
**(A)** Principal component analysis of the metabolite profile of the greenhouse (PPR) and natural forest plant samples (LPPR). The variance of each component is indicated on the plot in percentages. **(B)** OPLS-DA analysis for the detected compounds in the roots of the artificially grown greenhouse plants and naturally grown *P. polyphylla* plants.

The main purpose of the study was to evaluate the changes in the metabolites induced by different growth conditions, which could enhance our understanding of the differences in their medicinal values. A total of 1,182 metabolites were detected based on the UPLC-MS/MS detection platform and self-built database. The PCA score plots were visualized for the annotated dataset of the metabolites (with three replicates in each group). The PCA score plots for the greenhouse (PPR1-3) and forest samples (LPPR1-3) are shown in a 2D PCA plot (Figure 4). Environment-dependent separation was observed in all samples (Figure 4). These results suggested that the variation in the metabolites could be attributed to the differences in the growth conditions rather than their genetic backgrounds. Subsequently, we performed the orthogonal projections to latent structures discriminant analysis (OPLS-DA) to further highlight the dissimilarities between the two groups. Indeed, the OPLS-DA analysis showed a clear separation between the PPR and LPPR samples across all time intervals (Figure 4B).

**Figure 5:**
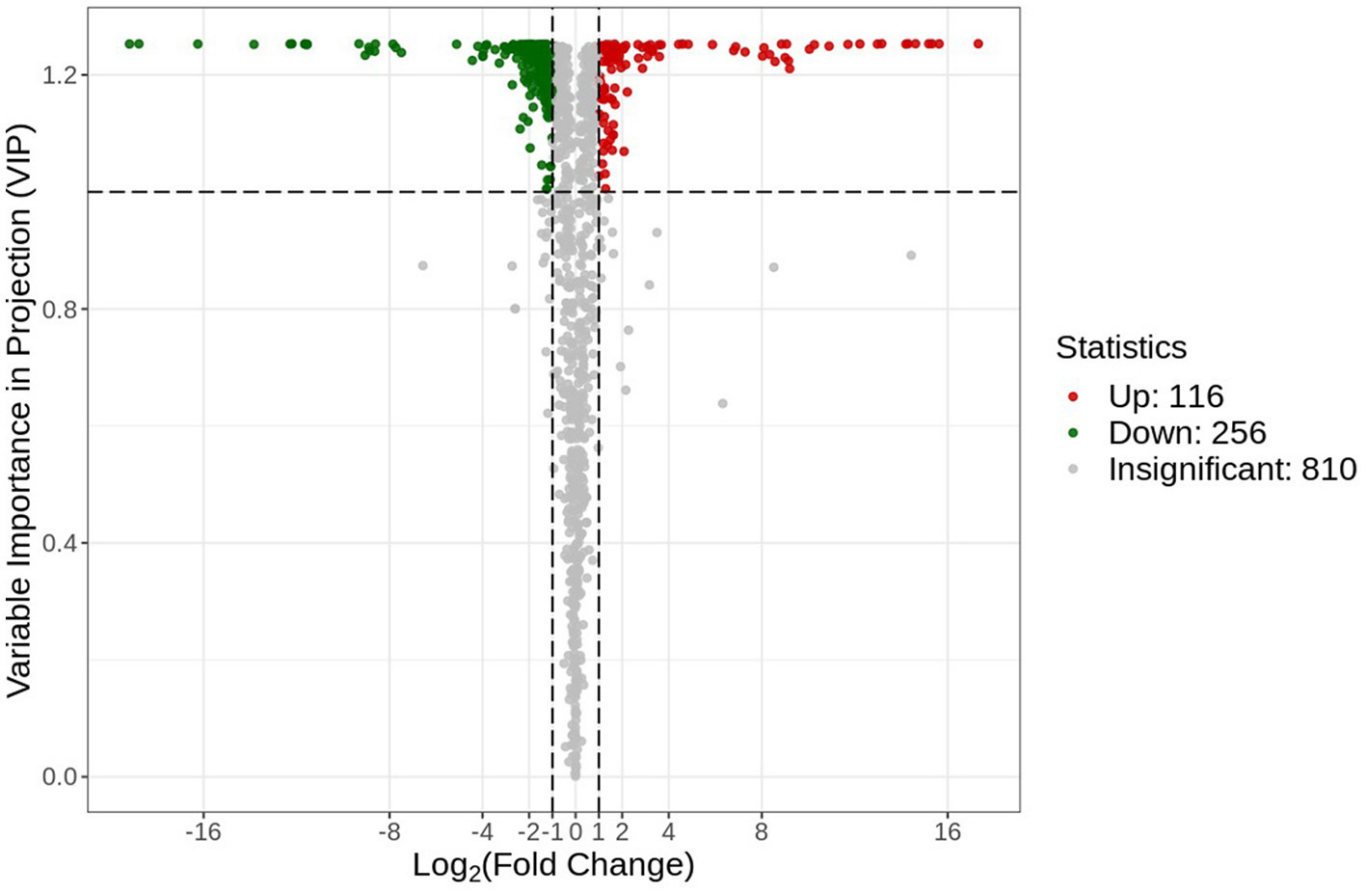
Differential metabolite volcano plot. Each point in the plot represents a metabolite, where the green points represent the downregulated differential metabolites, the red points represent the up-regulated metabolites, and the gray points represent the metabolites that were non-significantly different.

A volcano plot analysis was performed to estimate the relative difference in the number of metabolites that were significantly accumulated or reduced (*p* < 0.05) in the roots of both groups (Figure 5). The volcano plot analysis revealed three different levels of metabolite accumulation. The green points represent the lower relative contents (downregulated = 256), while the red points represent the upregulated metabolites (upregulated = 116). Statistically, downregulated and upregulated metabolites can be observed by their position at the vertical axis. This finding indicated a significant change at the metabolomic level for the plants in both environmental conditions. The gray points in the volcano plot represent those types of detected metabolites that did not meet the statistically significant threshold level. These metabolites were either in small quantities or at moderate differences in the relative content between the two groups.

### 3.3 Differentially accumulated metabolites in root tissues

**Figure 6:**
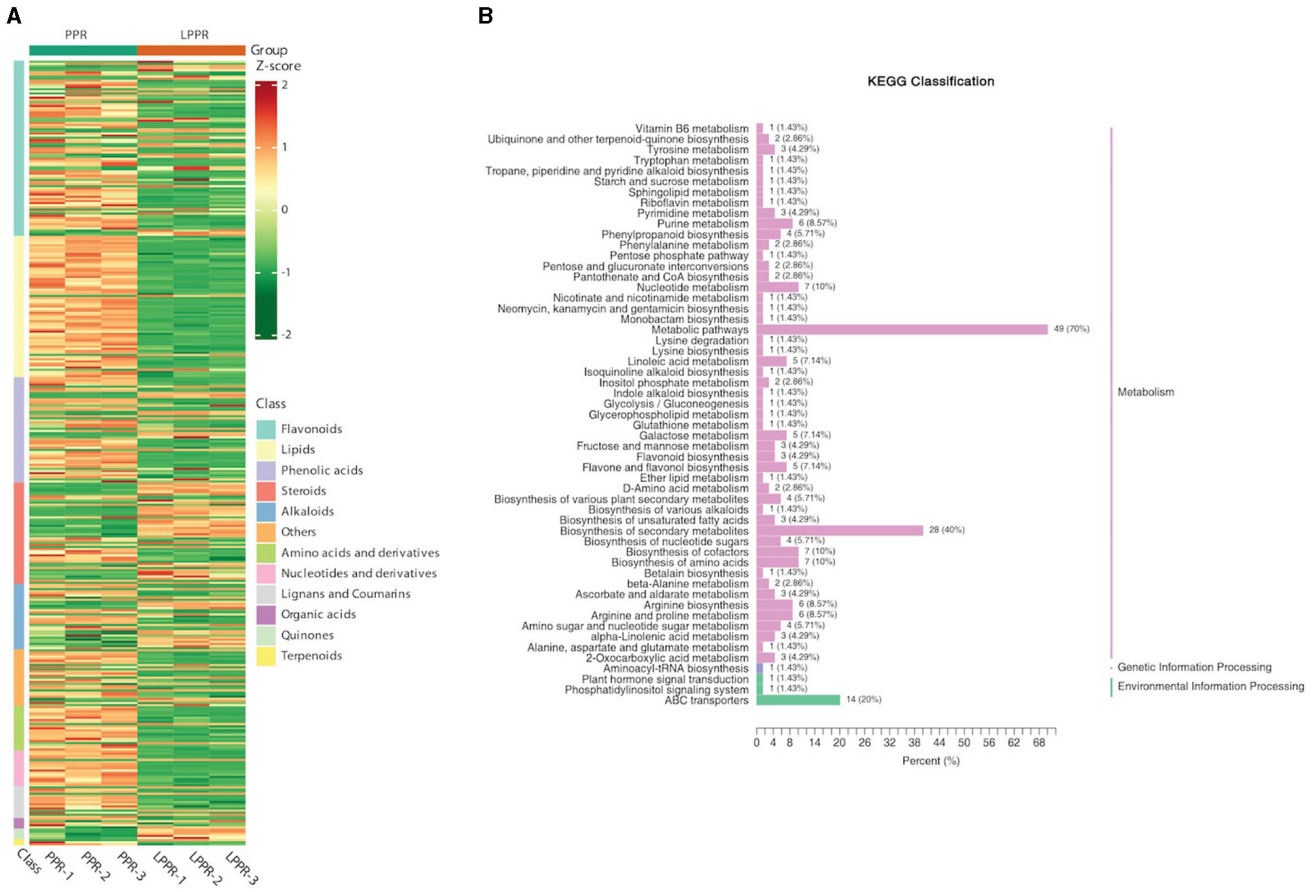
**(A)** Overall heatmap cluster diagram. The different colors in the heatmap represent different values obtained after the normalization of different relative contents. Different classes of the metabolites are represented on the right side of the heatmap diagram. **(B)** Metabolites enriched in the KEGG pathway: The ordinate names of the KEGG metabolic pathways are mentioned on the left side, while the ratio of their number to the total number of the differentially annotated metabolites is shown on the right side.

The UPLC-MS/MS analysis determined the composition of metabolites in the roots of *P. polyphylla* plants in the greenhouse and maintained naturally. As shown in the PCA analysis, significant metabolic differences were observed between both groups. Overall, 372 metabolites were separated based on their differential regulation levels (Supplementary File S2). To better understand the chemical differences between the greenhouse and naturally maintained *P. polyphylla* plants, a cluster heatmap of six samples was plotted (Figure 6A). By matching these differential metabolites in the database, we performed the cluster analysis on both metabolites and samples. The heatmap results showed homogeneity of the samples within the groups, while high heterogeneity was observed between the two groups (Figure 6A).

**Figure 7:**
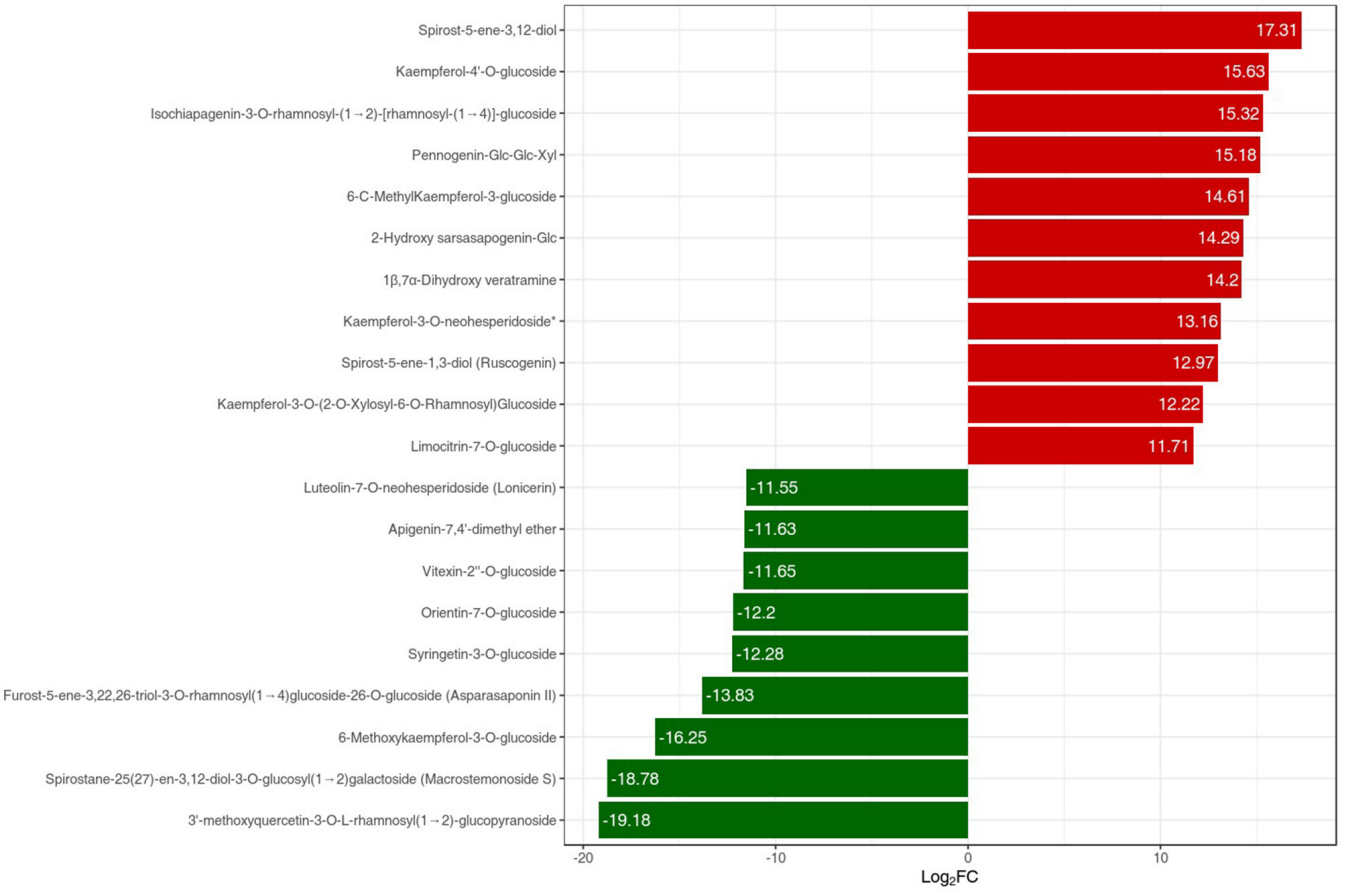
PPR vs. LPPR bar chart index of the top 20 differentially expressed metabolites. The red color bar represents an increase in the metabolite content, while the green color bars represent a decrease in the metabolite content.

The metabolites interacting with each other were annotated using the KEGG database, and only the differentially accumulated metabolic pathways were annotated. The annotation results of the differential metabolites were classified according to the pathway types (Figure 6B). We identified that the differentially accumulated metabolites were enriched in the naturally grown *P. polyphylla* plants. These enriched pathways included the biosynthesis of secondary metabolites and certain other metabolic pathways. The overall analysis indicated a higher accumulation of flavanols and steroids in the naturally maintained plants. For example, the flavonoids-I biosynthesis pathway [6,8-Dihydroxy-2-(2-hydroxy-4-methoxybenzyl)-7-methyl-3,4-dihydronaphthalen-1(2H)-one] was upregulated. Similarly, certain other steroids, namely, *Steroidal saponins* (14-Dehydroxy-neoprazerigenin A-Glc-Glc-Rha-Rha), were also found to be upregulated in the root tissues of the naturally grown *P. polyphylla* plants. Other than flavonoids and steroids, certain other vitamins were also enriched in the naturally grown *P. polyphylla* plants as compared to the greenhouse plants. After the qualitative and quantitative analyses of the detected metabolites, we compared the top 20 metabolites with the highest fold difference in each group (Figure 7). The bar chart for the top 10 upregulated compounds indicated the enrichment of the steroidal saponins (spirost-5-ene-3,12-diol and spirost-5-ene-1,3-diol) and flavonoid compounds such as kaempferol-3-O-neohesperidoside.

## 4 Discussion

*P. polyphylla* is a perennial herb belonging to the Trilliaceae family and grows well in the temperate forests of southwest China. Its rhizome is locally known as “Chonglou” and is considered an integral component of traditional Chinese medicine (TCM). It is traditionally used in a powder or tablet form for treating swelling, throat infections, body pains, and tumors (Jing et al., 2016). Due to its medicinal importance, a series of experiments have been conducted to characterize the extracts of PPs (Shen et al., 2018; Qin et al., 2012; Wei et al., 2014). The chemical composition of its extract contains a large number of flavonoids, steroidal compounds, and amino acids (Yue et al., 2021; Pei et al., 2018). *P. polyphylla* plants are slow-growing plants and are in great demand by the drug industry. Due to their extensive use, the natural sources of Chonglu are depleting. Moreover, it has been reported that only 40% of *P. polyphylla* seeds can germinate under natural conditions (Deb et al., 2015). As an alternative, its aboveground parts, such as leaves and stems, have also been studied (Qin et al., 2018). In addition, the establishment of callus cultures for *P. polyphylla* has been introduced to extract secondary metabolites for commercial applications (Rawat et al., 2023).

Understanding the differences in the accumulation of metabolites is important for the pharmaceutical industry. The present study was conducted to explore the metabolome of *P. polyphylla* in both naturally and artificially maintained conditions. For exploring the composition of metabolites in both conditions, the widely adopted ultra-performance liquid chromatography was used. The identified metabolites were divided into 12 broad categories, including alkaloids, amino acids and derivatives, flavonoids, steroids, and terpenoids. Although the metabolites of *P. polyphylla* are already known, previous studies have not demonstrated the enrichment of medically important compounds in comparison to artificial and natural conditions. In the differential analysis, we identified 372 compounds from different fractions (Supplementary material), including 83 flavonoids, 67 lipids, 48 steroids, 15 lignans and coumarins, 5 quinones, and 3 terpenoids. A variable proportion of vitamins, alkaloids, amino acids, nucleotides derivatives, and organic acids was also identified. The extracts from the forest-grown *P. polyphylla* plants showed a significantly higher content of total phenols, flavonols, and flavonoids. In previous studies, these compounds were shown to have inhibitory effects on cancer cells (Lepcha et al., 2019). We identified differential upregulation of spirost-5-en-3,12-diol in the wild plants, which is a naturally existing antioxidant steroid potentially used to treat neurodegenerative diseases (Jesus et al., 2016). In a previous study, spirost-5-en-3,12-diol was shown to exist in *Smilax China* and *Trigonella foenum* (Jesus et al., 2016). The list of the top 10 upregulated metabolites also included kaempferol, which is another important flavonoid used as an antioxidant, anti-inflammatory, and antimicrobial product. Studies have also revealed that kaempferol harbors reactive oxygen species (ROS)-scavenging properties (Chen et al., 2013). In the present study, the data indicated the abundance of steroidal saponins in the naturally grown *P. polyphylla* plants as compared to the greenhouse plants. As sapogenins are valuable compounds for the pharmaceutical industry due to the wide spectrum use as cardioprotective agents and cAMP phosphodiesterase inhibitors, there is a dire need to protect natural resources such as *P. polyphylla* plants.

The changes in the lipid metabolites were also strongly correlated with the naturally grown plants compared to the greenhouse plants. Previous studies have demonstrated that phospholipases were upregulated during plant germination through seeds (Tang et al., 2022). In our experiments, the higher accumulation of lipids in the naturally grown plants demonstrated the storage of lipids and their precursors in the roots. The increase in the accumulation of fatty acids provides a clue about their adaptive fitness for improved germination under natural conditions. Another explanation for differential metabolite accumulation can be attributed to the natural germination process of the seeds of *P. polyphylla* plants after long dormancy. The transition of seeds from dormancy to germination may induce epigenetic changes in the physiology of *P. polyphylla* plants (Jiahui et al., 2024), which may ultimately lead to differences in the accumulation of metabolites.

## 5 Conclusion

Through the metabolomics analysis, we investigated the differential accumulation of metabolites in naturally occurring *P. polyphylla* and artificially maintained plants. We demonstrated that, although artificially maintained *P. polyphylla* plants can be a reliable source of flavonoids and naturally occurring steroids of medicinal importance, plants grown under natural conditions show an increased level of secondary metabolites, including flavonoids, flavonols, and natural steroids. Our analysis supports the concept that the type and concentration of secondary metabolites produced in a plant are determined by the environmental conditions during growth. Therefore, the choice of medicinally important components should be based on the environmental conditions of *P. polyphylla*.

## Data Availability

The data presented in the study are deposited in the National Genomics Data Center repository, accession number PRJCA030775 (https://ngdc.cncb.ac.cn/bioproject/browse/PRJCA030775).
