# Supplementary material for: Understory growth of Paris polyphylla accumulates a reservoir of secondary metabolites of plants
Source: Front Microbiol. 2024 Oct 15;15:1400616. doi: 10.3389/fmicb.2024.1400616 (PMC11518744; doi:10.3389/fmicb.2024.1400616)
Supplement: Supplementary file 1 [file Data_Sheet_1.docx]

Supplementary Material


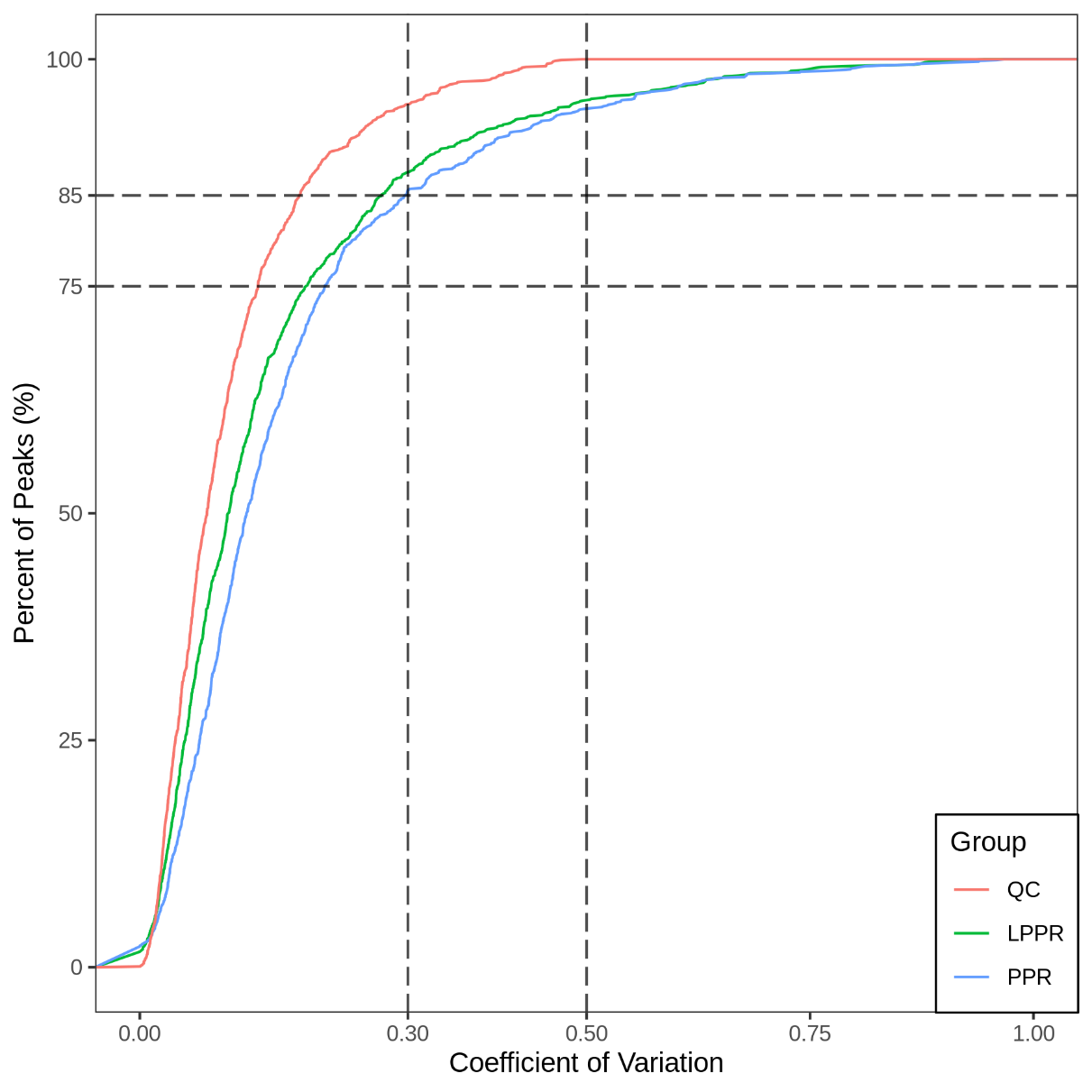


**Supplementary Figure 1.**: CV distribution chart of each group of samples

Note: The abscissa represents the CV value, the ordinate represents the proportion of the number of substances smaller than the corresponding CV value to the total number of substances, different colors represent different grouped samples, QC is the quality control sample, and the two reference lines perpendicular to the X-axis correspond to The CV values ​​are 0.3 and 0.5, and the two reference lines parallel to the X-axis correspond to 75% and 85% of the total number of substances.

**Supplementary file-2**. List of 372 differentially accumulated metabolites in root tissues of *Paris polyphylla* samples (MS Excel file attached).
